# Protocol for the GOLF trial: randomized clinical trial on the LINX management system *versus* fundoplication for the surgical treatment of gastro-oesophageal reflux disease

**DOI:** 10.1093/bjs/znaf141

**Published:** 2025-07-12

**Authors:** Sheraz R Markar, Begum Zeybek Saglam, Nainika Menon, Ahmed Ahmed, Nick Maynard, James Gossage, Filipa Landeiro, Jane Blazeby, Nicola Mills, Tim Underwood, Mimi McCord, T Martyn Hill, Amy Taylor, Natalie Blencowe, Jesper Lagergren

**Affiliations:** Surgical Intervention Trials Unit, Nuffield Department of Surgical Sciences, University of Oxford, Oxford, UK; Surgical Intervention Trials Unit, Nuffield Department of Surgical Sciences, University of Oxford, Oxford, UK; Surgical Intervention Trials Unit, Nuffield Department of Surgical Sciences, University of Oxford, Oxford, UK; Department of Surgery and Cancer, Imperial College London, London, UK; Department of Oesophago-Gastric Surgery, Oxford University Hospitals NHS Foundation Trust, Oxford, UK; Department of Surgery, Guy’s and St Thomas’ NHS Foundation Trust, London, UK; School of Cancer and Pharmaceutical Sciences, King’s College London, London, UK; Health Economics Research Centre, University of Oxford, Oxford, UK; NIHR Bristol Biomedical Research Centre and Centre for Surgical Research, Population Health Sciences, University of Bristol, Bristol, UK; Bristol Medical School, Population Health Sciences, University of Bristol, Bristol, UK; Gastrointestinal Surgery, University of Southampton, Southampton, UK; Heartburn Cancer UK Charity, UK; Surgical Intervention Trials Unit, Nuffield Department of Surgical Sciences, University of Oxford, Oxford, UK; Surgical Intervention Trials Unit, Nuffield Department of Surgical Sciences, University of Oxford, Oxford, UK; NIHR Bristol Biomedical Research Centre and Centre for Surgical Research, Population Health Sciences, University of Bristol, Bristol, UK; Department of Surgery, Guy’s and St Thomas’ NHS Foundation Trust, London, UK; School of Cancer and Pharmaceutical Sciences, King’s College London, London, UK

## Introduction

Gastro-oesophageal reflux disease (GORD) represents a significant burden on the Western healthcare system, affecting up to 20% of adults, with a rising prevalence^1,2^. GORD negatively impacts a patient’s health-related quality of life (HRQoL) and is associated with an increased risk of complications, including inflammation and strictures, Barrett’s oesophagus, and oesophageal adenocarcinoma^3^. Long-term use of proton pump inhibitors (PPIs) remains the mainstay of medical treatment for GORD; however, these may be associated with an increased risk of side effects, including dementia, renal pathology, infections, fractures, and gastric cancer^4^. A large UK RCT (REFLUX) showed that surgery (laparoscopic fundoplication) offers the most effective symptom control at 5-year follow-up, as well as being the most cost-effective treatment strategy when compared with medical therapy^5,6^.

Laparoscopic fundoplication is currently the ‘gold standard’ surgical treatment for managing GORD, with an excellent safety profile and a 30-day mortality risk of 0.03%^7^. The side effects are mainly gas bloating and inability to belch (up to 85%), dysphagia (3–24%), diarrhoea (18–33%), and recurrence of reflux symptoms (10–62%)^8,9^. Approximately 5% of patients undergoing fundoplication in England may require secondary surgery and 60% of patients use antireflux medication within 12 months of primary antireflux surgery^7^.

The introduction of the LINX device in 2007 provided a surgical alternative to fundoplication, requiring less extensive dissection and less disruption of the hiatal anatomy and natural antireflux mechanisms^10,11^. The LINX device is placed around the distal oesophagus and consists of titanium beads with a magnetic core that augments lower oesophageal tone and thus prevents reflux by mimicking normal anatomical antireflux mechanisms^12^. The LINX device, while augmenting the lower oesophageal sphincter, can accommodate the escape of elevated gastric pressure associated with belching or vomiting, which may reduce gas bloating. Complications of the LINX device include dysphagia, requiring dilatation at the site of the device in 5–11% of patients, and endoluminal erosions (0.1%) requiring device removal^11,13^.

In non-randomized comparative studies patients have reported favourable outcomes with LINX compared with fundoplication^13^. Aside from its ease of insertion, the LINX device is appealing in terms of symptom control, shorter operating time, reduced hospital stay, and lower burden of postoperative care^14^. A systematic review and meta-analysis of the laparoscopic LINX procedure *versus* laparoscopic fundoplication (consisting of 6 cohort studies and 1099 patients) showed no statistically significant differences between the groups in the requirement of postoperative antireflux medication, GORD-HRQoL scores, dysphagia, or reoperation. However, LINX was associated with significantly less gas bloating (pooled OR 0.34 (95% c.i. 0.16 to 0.71)) and a greater ability to belch (pooled OR 12.34 (95% c.i. 6.43 to 23.7))^13^. A further systematic review evaluated the introduction of LINX in the context of the established Idea, Development, Exploration, Assessment, and Long-term follow-up (IDEAL) framework^15^. Several IDEAL phase IIb studies were identified, with a lack of standardized surgical quality assurance (SQA) regarding LINX implantation and lack of consensus regarding results that should be evaluated to meaningfully assess patient benefit. This review concluded that studies that are well designed and well conducted are needed to evaluate the LINX procedure.

Although the National Institute for Health and Care Excellence (NICE) allows the use of the LINX device in clinical practice, it encourages research in this area, particularly trials that compare the LINX device with other forms of antireflux surgery^16^. The aim of this RCT is to test the hypothesis that the LINX procedure achieves similar reflux control and improves postoperative symptoms, specifically gas bloating and inability to belch, when compared with fundoplication at 24 months after surgery.

## Methods

### Design

**Fig. 1:**
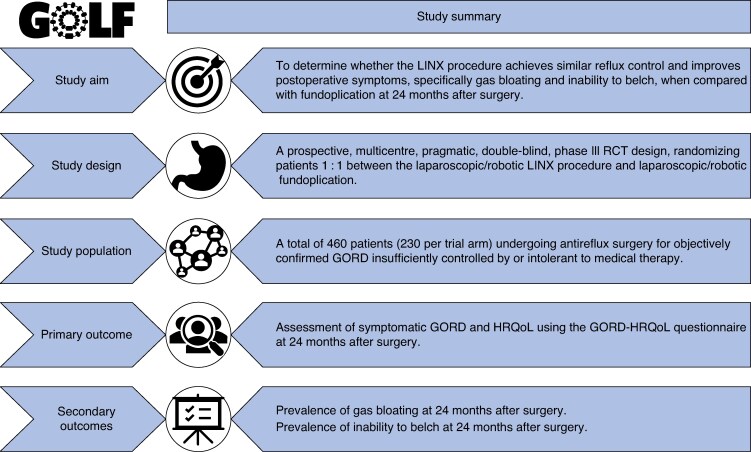
Study summary flow chart GORD, gastro-oesophageal reflux disease; HRQoL, health-related quality of life.

**Fig. 2:**
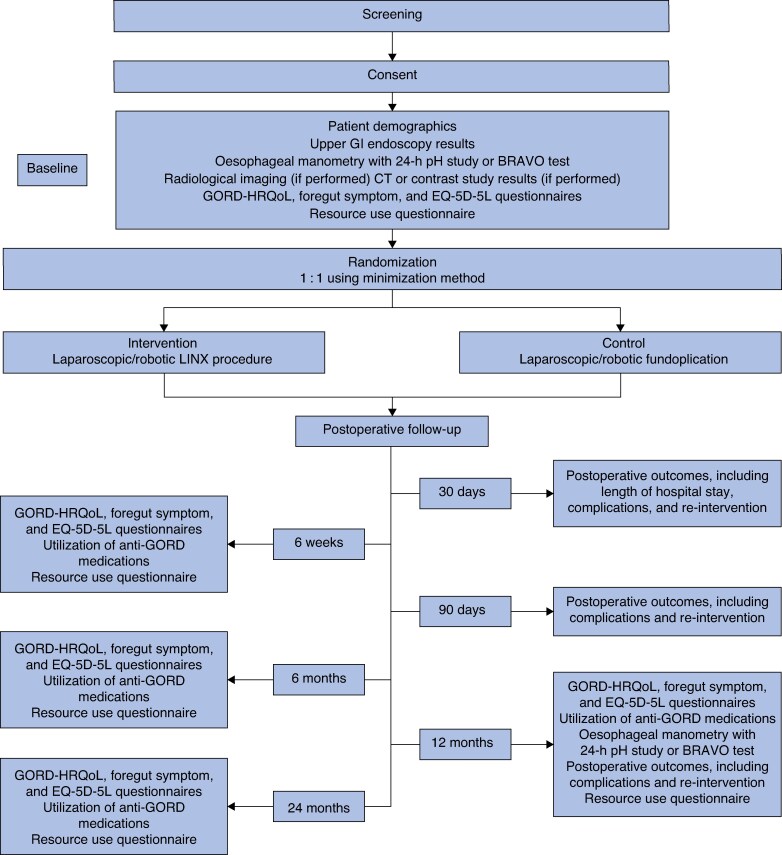
Data collection flow chart GI, gastrointestinal; GORD, gastro-oesophageal reflux disease; HRQoL, health-related quality of life.

**Fig. 3:**
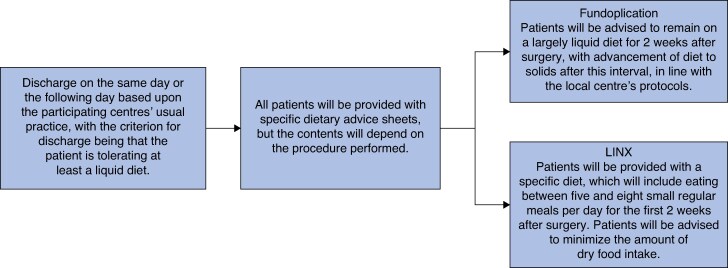
Post-study care and follow-up flow chart

The GOLF trial is an international, multicentre, pragmatic, two-arm, double-blind, phase III RCT. The study will recruit 460 patients (230 patients in each of the two arms) recommended for antireflux surgery from at least 16 centres in the UK and 7 other European high-volume upper gastrointestinal surgical centres. Patients will be randomized 1 : 1 to undergo either a laparoscopic/robotic LINX procedure or fundoplication. See *Fig. 1* for the study summary flow chart, *Fig. 2* for the data collection flow chart, and *Fig. 3* for the post-study care and follow-up flow chart.

### Population

The GOLF trial will recruit adults aged ≥18 years being considered for antireflux surgery for GORD insufficiently controlled by or intolerant to medical therapy. The inclusion and exclusion criteria are presented in *Table 1*.

**Table 1:** Inclusion and exclusion criteria for the GOLF trial

Inclusion criteria	Exclusion criteria
Age ≥18 years Willing and able to give informed consent GORD insufficiently controlled by medical therapy or intolerance to medical therapy and being considered for antireflux surgery Symptomatic and objectively defined GORD; endoscopy with appearances or biopsies consistent with reflux oesophagitis, or 24-h pH study or BRAVO test of the oesophagus consistent with GORD No hiatal hernia or hiatal hernia <5 cm in length Adequate lower oesophageal motility as defined by preoperative oesophageal manometry studies showing a mean contractile amplitude of >30 mmHg or distal contractile integral (DCI) of >450 mmHg per s per cm in 70% of swallows	Unsuitable for surgical intervention due to medical conditions precluding general anaesthesia Suspected or known allergies to titanium, stainless steel, nickel, or ferrous materials Previous antireflux or gastric surgery Previous or planned neurosurgery Oesophageal manometry showing complete absence of lower oesophageal contractility

GORD, gastro-oesophageal reflux disease.

#### Screening and recruitment

Participants will be recruited from hospitals in the UK and other European countries that regularly perform antireflux surgery. Potential participants will be identified during routine clinic visits and informed about the study through posters and Participant Information Sheets (PISs). If their usual care clinician is not part of the study team, patients will be asked for permission to share their contact details with the study team for follow-up.

#### QuinteT Recruitment Intervention (QRI)

The LINX procedure is not as well established as fundoplication. Recruitment is likely to be challenged by clinician and patient bias towards something new, potentially impacting eligibility decisions and how the treatments are presented. Furthermore, some centres may be less familiar explaining randomization to patients. A QRI has been included to understand and address recruitment challenges in real time^17–19^. The aim of the QRI is to assess the recruitment process at each recruiting site, to identify specific recruitment difficulties and tailor suggestions to change aspects of the design, conduct, or training that could lead to improvements in recruitment and informed consent.

A multifaceted, flexible approach will be used to investigate site-specific or wider recruitment obstacles, consisting of: mapping of eligibility and recruitment pathways to collate basic data about the levels of eligibility and recruitment, and to identify bottlenecks in recruitment pathways; in-depth semi-structured interviews with study and centre staff (and eligible patients if needed) to explore perceptions of the study and equipoise, how recruitment is organized in individual centres, and local or national challenges anticipated or encountered; audio-recording study recruitment discussions with potential study participants to offer direct insights into how the study is presented and ways in which recruiters manage patients’ expectations and preferences; and attendance at study meetings to gain an overview of trial conduct and overarching challenges.

The QRI team, with the Chief Investigator and Trial Management Group, will formulate action plans based on the findings to improve recruitment and information provision, with the format dependent on the nature of the recruitment barriers identified. Supportive and responsive group or individual feedback and training is likely to be a core component of the plan of action, including written recruitment ‘tips’ documents and suggested modifications to study pathways and patient-facing study material. The QRI work will be undertaken in an iterative and cyclical manner, continuing throughout the recruitment interval with close monitoring of changes in screening log data and recruiter practice to optimize recruitment and informed consent, all in close collaboration with the Chief Investigator and wider study team.

#### Laparoscopic or robotic LINX procedure (intervention)

The laparoscopic insertion of a magnetic ring, such as the LINX device, has been part of the NICE guidelines for treating GORD and therefore standard of care in the National Health Service (NHS) since 2017. Participants randomized to laparoscopic or robotic magnetic sphincter augmentation (LINX procedure) will undergo surgical treatment under general anaesthesia, with placement of the LINX device around the distal oesophagus. The LINX device consists of titanium beads with magnets in the centre.

#### Laparoscopic or robotic fundoplication (comparator)

Participants randomized to laparoscopic or robotic fundoplication will undergo surgical treatment for managing GORD, including a total or partial fundic wrap behind or in front of the distal oesophagus and gastro-oesophageal junction. According to several international guidelines, there is no convincing evidence at present to suggest a total or partial wrap to be superior to the other. As this is a pragmatic trial, all types of fundoplication will be recorded and form part of the subgroup analysis^8,20,21^.

#### Translational ‘SQA’ substudy

SQA is an important component of this trial because LINX is a newer surgical procedure and because there is (inter)national variation in fundoplication techniques. This is the first trial of its kind to embed an SQA programme based on video analysis. The SQA will help to ensure the procedures are also completed to a good surgical standard, to maximize internal and external validity and facilitate accurate interpretation of trial results and replication of the successful intervention across wider clinical practice. Every operation performed within the trial will be video recorded and reviewed as part of the SQA programme.

The SQA programme will consist of five phases:

- Surgeon training: Surgeons who have not previously undertaken the LINX procedure will attend a hands-on training day with Johnson and Johnson, followed by two proctored cases. A ‘gold standard’ demonstrative video will be circulated to all participating surgeons along with the competency assessment tools for the LINX procedure and fundoplication to ensure consistency.
- Credentialling of centres and surgeons: All surgeons who have performed <20 LINX procedures will be required to submit two videos each of them performing the LINX procedure and fundoplication, which will be assessed using a competency assessment tool (described below in phase 3).
- Standardization of surgical techniques: A competency assessment tool for the LINX procedure will be developed after a virtual Delphi consensus process and robust testing for inter- and intra-assessor reliability for video assessment. The Society of American Gastrointestinal and Endoscopic Surgeons (SAGES) has developed and validated a competency assessment tool for fundoplication, which will be used in the GOLF trial^22^.
- Mechanistic work: Video assessment will be undertaken for all operations performed within the trial to monitor learning curves and maintain surgical quality.
- Ongoing monitoring of adherence to the intervention protocols: This will be associated with correlating surgical quality with clinical and patient-reported outcomes.

### Outcomes

#### Primary outcome

Symptomatic reflux and HRQoL assessed using the GORD-HRQoL questionnaire^23^ at 24 months after surgery (*Table 2*).

**Table 2:** GERD-HRQoL questionnaire

Please check the number that best reflects your symptoms using the scoring scale provided below	
0 = No symptoms	3 = Symptoms bothersome every day
1 = Symptoms noticeable but not bothersome	4 = Symptoms affect daily activities
2 = Symptoms noticeable and bothersome but not every day	5 = Symptoms are incapacitating—unable to do activities

Check only one box for each question						
How bad is your heartburn?	□ 0	□ 1	□ 2	□ 3	□ 4	□ 5
Heartburn when lying down?	□ 0	□ 1	□ 2	□ 3	□ 4	□ 5
Heartburn when standing up?	□ 0	□ 1	□ 2	□ 3	□ 4	□ 5
Heartburn after meals?	□ 0	□ 1	□ 2	□ 3	□ 4	□ 5
Does heartburn change your diet?	□ 0	□ 1	□ 2	□ 3	□ 4	□ 5
Does heartburn wake you from sleep?	□ 0	□ 1	□ 2	□ 3	□ 4	□ 5
Do you have difficulty swallowing?	□ 0	□ 1	□ 2	□ 3	□ 4	□ 5
Do you have bloating or gassy feelings?	□ 0	□ 1	□ 2	□ 3	□ 4	□ 5
Do you have pain with swallowing?	□ 0	□ 1	□ 2	□ 3	□ 4	□ 5
If you take medication, does this affect your daily life?	□ 0	□ 1	□ 2	□ 3	□ 4	□ 5
How satisfied are you with your present condition?	□ Satisfied		□ Neutral		□ Dissatisfied	

GORD, gastro-oesophageal reflux disease; HRQoL, health-related quality of life.

#### Secondary outcomes

- Prevalence of gas bloating measured using participant-reported outcomes/GORD-HRQoL and foregut symptom questionnaire^24^ at 24 months after surgery.
- Prevalence of inability to belch measured using participant-reported outcomes/GORD-HRQoL and foregut symptom questionnaire at 24 months after surgery.
- Prevalence of reflux symptoms, inability to belch, and gas bloating measured using participant-reported outcomes/GORD-HRQoL and foregut symptom questionnaire at 6 weeks and 6 and 12 months after surgery.
- Prevalence and severity of dysphagia and regurgitation measured using participant-reported outcomes/GORD-HRQoL questionnaire at 6 weeks and 6, 12, and 24 months after surgery.
- Global HRQoL measured using participant-reported outcomes/EQ-5D-5L^25^ questionnaire at 6 weeks and 6, 12, and 24 months after surgery.
- Utilization of antireflux medications measured using participant-reported outcomes/questionnaire at 6 weeks and 6, 12, and 24 months after surgery.
- 24-h pH measurement or BRAVO test at 12 months after surgery.
- 30- and 90-day and 12- and 24-month postoperative complication rates, including reoperation and endoscopic re-intervention, measured using participant’s medical records/postoperative outcomes.
- Cost-effectiveness measured using incremental cost per quality-adjusted life year (QALY)^26^ at 6 weeks and 6, 12 and 24 months after surgery.

The total duration of trial recruitment is expected to be 30 months. Patients will be followed up for 24 months after surgery. The GOLF trial will be embedded within the Association of Upper Gastrointestinal Surgeons for Great Britain and Ireland (AUGIS) benign surgical registry, which will permit follow-up reports at 5 and 10 years after surgery. Patient consent will also be obtained to link their data and ensure comprehensive follow-up to assess the long-term outcome and safety of the LINX procedure compared with fundoplication.

### Sample size

Sample size calculations were based on a hierarchical analysis and non-inferiority between the study arms for HRQoL including control of reflux (primary outcome) and superiority in favour of the LINX procedure for gas bloating and inability to belch (core secondary outcomes).

#### Non-inferiority outcome

A non-inferiority margin was set at two scores in difference on GORD-HRQoL based on a previous systematic review by the authors^13^, co-investigator consensus, and patient workshops. For a one-sided α level of 0.025 and expecting 10% loss to follow-up, 230 patients per group will be necessary to show non-inferiority with 90% power.

#### Superiority outcomes

A two-sided α level of 0.050 and 10% loss to follow-up were assumed for the superiority outcomes. Regarding postoperative gas bloating, meta-analysis prevalence’s estimate of postoperative gas bloating of 30.1% for fundoplication and a meta-analysis of efficacy showed an OR of 0.34 in favour of LINX^13^, which correspond to a 12.7% prevalence of gas bloating for LINX. Thus, a reduction in postoperative gas bloating of 17.4% (from 30.1% to 12.7%) is hypothesized. According to those parameters, 144 patients per group will be necessary to verify that change with 90% power.

### Randomization

Randomization will be performed using a minimization algorithm to ensure balance between the two treatment groups using stratification factors:

- Age (<40, 40–60, and >60 years).
- Sex at birth (female and male).
- Co-morbidity at baseline according to the well-validated Charlson Co-morbidity Index (<2 and ≥2).
- BMI (<30 and ≥30 kg/m^2^).
- Preoperative DeMeester score (a composite score system of acid exposure during ambulatory pH monitoring used to objectively define GORD) (<14.7 and ≥14.7).
- Country of treatment (UK and non-UK).
- 24 months after the surgery, patients will be unblinded (that is informed via letter or e-mail about which operation they underwent) and then they will be followed up according to the standard of care in their treating hospital.

### Blinding

This study will be double-blinded (that is the patients and the outcome assessors will be blinded to the trial arms). All postoperative symptomatic questionnaires will be collected electronically directly from blinded patients or by telephone interviews conducted by blinded research nurses and/or blinded central study management staff. At 12 months after surgery, patients will undergo a 24-hour pH study or BRAVO test, as well as manometry investigations, preformed by blinded assessors. Blinding effectiveness will be evaluated for both patients and outcome assessors using the Bang Blinding Index (BBI)^27^. Surgeons and registrars will not be blinded due to the nature of the surgical intervention. The surgeons and registrars will be responsible for adding the patient to the AUGIS benign surgical Registry, which will only be used for follow-up beyond 2 years, when the patients will be unblinded. After randomization, an e-mail confirming treatment allocation will be sent to the principal investigator (unblinded only for participants they randomize) and the delegated operating surgeon. In the event of a serious adverse event, additional site staff may be unblinded if necessary to ensure patient safety.

### Statistical analysis

All analyses will be carried out on the intention-to-treat population (that is all patients will be analysed in the group that they were randomized to regardless of the actual treatment received). It is not anticipated that there will be any protocol deviations; however, in the event that any occur, the primary analysis will be repeated for the per-protocol population (patients excluded from the per-protocol population will be pre-specified in the Statistical Analysis Plan).

Standard descriptive statistics will be used to describe the demographics between the two follow-up regimens; reporting means and standard deviations or medians and interquartile ranges as appropriate for continuous variables and numbers and percentages for binary and categorical variables. All comparative outcomes will be presented as summary statistics and reported together with 95% confidence intervals. All tests will be carried out at a 5% significance level.

The analysis of GORD-HRQoL will be performed using a linear mixed model. This model will produce as output an adjusted GORD-HRQoL score based on the fixed and random effects. It will consider, as the fixed effects, the intervention group, as well as the age, sex, BMI, and preoperative DeMeester score. It will consider, as the random effects, a random intercept by centre. An interaction between the random intercept centre and the intervention will also be included in the model. The model will be run and the output adjusted GORD-HRQoL scores will be compared.

The effect of the LINX procedure on GORD-HRQoL will be tested and quantified through mean differences between groups, adjusted for the factors included in the model; the 97.5% unilateral confidence interval of the mean differences will be provided. A transformation of the primary criterion may be performed to fulfil the assumptions of the linear mixed model.

### Health economic analysis

A within-trial analysis will be conducted to assess the cost-effectiveness of laparoscopic LINX compared with fundoplication. An NHS and Personal Social Services perspective will be used for the base-case analysis and a societal perspective will be presented in the sensitivity analysis. The primary outcome measure used in the health economics study will be incremental cost per QALY. Good practice guidelines will be followed when undertaking the economic evaluation analysis.

A resource use questionnaire will be used to collect all healthcare (primary care appointments, prescribed and over the counter medications, hospital admissions, contact with other healthcare professionals) and non-healthcare (time off work) resource use of patients undergoing any of the procedures assessed in the trial. The questionnaire will be administered to patients at baseline and at 6 weeks and 6, 12, and 24 months after surgery. The resources used will be valued using national cost databases, such as NHS Reference Costs and Prescription Cost Analysis.

The EQ-5D-5L instrument will be used to measure HRQoL at baseline and at 6 weeks and 6, 12, and 24 months after surgery. The EQ-5D-5L instrument will be valued using NICE recommendations at the time of the analysis, using a UK value set or converted into the EQ-5D-3L with a cross-mapping algorithm and valued using the UK set for EQ-5D-3L. QALYs will be calculated using the area under the curve approach, which involves estimating the average EQ-5D utility between each follow-up time and weighting it by survival time. Descriptive statistics (means and standard deviations as a minimum) will be reported for resource use, costs, and EQ-5D utilities at each follow-up time point. The baseline differences in resource use and utilities between the trial arms will be described and these differences will be adjusted for using the most appropriate recommended method. All costs and effects will be discounted at 3.5% following NICE guidelines. Best practice methods will be followed for addressing missing data in cost-effectiveness studies. Missing data on participant characteristics at baseline will be imputed following guidelines.

## Data Availability

Further data can be shared upon request from the corresponding author.
